# Exploring the Genetic Link Between Thyroid Dysfunction and Common Psychiatric Disorders: A Specific Hormonal or a General Autoimmune Comorbidity

**DOI:** 10.1089/thy.2022.0304

**Published:** 2023-02-14

**Authors:** Sourena Soheili-Nezhad, Emma Sprooten, Indira Tendolkar, Marco Medici

**Affiliations:** ^1^ Department of Cognitive Neuroscience, Donders Institute for Brain, Cognition and Behaviour, Radboud University Medical Centre, Nijmegen, the Netherlands.; ^2^ Language and Genetics Department, Max Planck Institute for Psycholinguistics, Nijmegen, the Netherlands.; ^3^ Department of Human Genetics, Radboud University Medical Centre, Nijmegen, the Netherlands.; ^4^ Department of Psychiatry, Donders Institute for Brain, Cognition and Behaviour, Radboud University Medical Centre, Nijmegen, the Netherlands.; ^5^ Department of Internal Medicine, Academic Center for Thyroid Diseases, Rotterdam, the Netherlands.; ^6^ Department of Epidemiology, Erasmus Medical Centre, Rotterdam, the Netherlands.; ^7^ Division of Endocrinology, Department of Internal Medicine, Radboud University Medical Centre, Nijmegen, the Netherlands.

**Keywords:** anxiety, autoimmune thyroiditis, bipolar disorder, depression, psychiatric comorbidity

## Abstract

**Background:**

The hypothalamus-pituitary-thyroid axis coordinates brain development and postdevelopmental function. Thyroid hormone (TH) variations, even within the normal range, have been associated with the risk of developing common psychiatric disorders, although the underlying mechanisms remain poorly understood.

**Methods:**

To get new insight into the potentially shared mechanisms underlying thyroid dysfunction and psychiatric disorders, we performed a comprehensive analysis of multiple phenotypic and genotypic databases. We investigated the relationship of thyroid disorders with depression, bipolar disorder (BIP), and anxiety disorders (ANXs) in 497,726 subjects from U.K. Biobank. We subsequently investigated genetic correlations between thyroid disorders, thyrotropin (TSH), and free thyroxine (fT4) levels, with the genome-wide factors that predispose to psychiatric disorders. Finally, the observed global genetic correlations were furthermore pinpointed to specific local genomic regions.

**Results:**

Hypothyroidism was positively associated with an increased risk of major depressive disorder (MDD; OR = 1.31, *p* = 5.29 × 10^−89^), BIP (OR = 1.55, *p* = 0.0038), and ANX (OR = 1.16, *p* = 6.22 × 10^−8^). Hyperthyroidism was associated with MDD (OR = 1.11, *p* = 0.0034) and ANX (OR = 1.34, *p* = 5.99 × 10^−^⁶). Genetically, strong coheritability was observed between thyroid disease and both major depressive (*r*_g_ = 0.17, *p* = 2.7 × 10^−^⁴) and ANXs (*r*_g_ = 0.17, *p* = 6.7 × 10^−^⁶). This genetic correlation was particularly strong at the major histocompatibility complex locus on chromosome 6 (*p* < 10^−^⁵), but further analysis showed that other parts of the genome also contributed to this global effect. Importantly, neither TSH nor fT4 levels were genetically correlated with mood disorders.

**Conclusions:**

Our findings highlight an underlying association between autoimmune hypothyroidism and mood disorders, which is not mediated through THs and in which autoimmunity plays a prominent role. While these findings could shed new light on the potential ineffectiveness of treating (minor) variations in thyroid function in psychiatric disorders, further research is needed to identify the exact underlying molecular mechanisms.

## Introduction

Thyroid hormone (TH) plays a key role in brain development and neurocognitive function.^1,2^ Both hypo- and hyperthyroidism have been associated with an increased risk of various psychiatric diseases,^3^ including depression, bipolar, and anxiety disorders (ANXs).^4^ More recently, it has even been shown that minor subclinical variations in thyroid function, even within the normal range, are associated with these psychiatric diseases,^5,6^ although some studies report contradicting findings.^7,8^ A better understanding of the biological processes underlying these associations could have highly valuable implications for both clinical psychiatry and endocrinology.

The most common forms of thyroid dysfunction have an autoimmune origin and there is increasing evidence that the immune system and inflammatory response are also relevant for the pathophysiology of psychiatric disorders.^9,10^ Next to the direct effects of TH on brain, the effects of thyroid dysfunction on psychiatric diseases could thus be mediated by more generally associated autoimmune processes.^11^ Various cellular mechanisms have been proposed for this phenomenon, ranging from binding of thyroid peroxidase (TPO) antibodies to human astrocytes, coinciding anticentral nervous system auto-antibodies in thyroid diseases, to increased production of monocyte- and T lymphocyte derived cytokines.^11^ *Vice versa*, psychiatric disorders have also been shown to be associated with abnormalities in thyroid function tests.^12,13^ These include mild elevations, as well as decreases in TH levels, blunting of the thyrotropin (TSH) response to thyrotropin-releasing hormone (TRH) stimulation, and absence of the nocturnal surge of TSH. In addition, psychiatric disorders have also been suggested to be associated to altered autoimmune processes.^14^

Taken together, there is still a large knowledge gap whether the observed associations between (minor or overt) thyroid abnormalities and psychiatric diseases are directly mediated through TH or not. This is a clinically relevant gap, as understanding the complex interactions between thyroid status, autoimmunity, and psychiatric disorders is a prerequisite before making treatment recommendations to prevent or treat coinciding psychiatric disorders in a patient with mild thyroid dysfunction or vice versa.

In the context of these complex interactions, it is noteworthy that an estimated 60–70% of the interindividual variation in thyroid function is determined by genetic factors.^15^ Next to a direct genetic relationship between thyroid function and psychiatric disorders, various studies have suggested that genetic factors play an important role in various other autoimmune diseases, as well as psychiatric disorders.^16–18^

In recent years, genome-wide association studies (GWASs) have been particularly successful in identifying the genetic determinants of thyroid function,^19,20^ and more recently of psychiatric disorders.^21^ With the availability of these data, this is the optimal moment to investigate the role of common genetic variants in the complex relationships between thyroid function, autoimmunity, and common psychiatric diseases. Doing so could help understand whether the well-known clinical comorbidity between thyroid diseases and psychiatric disorders is specifically driven by THs or has a more general autoimmune-related basis. Therefore, in the current study, we tested phenotypic and genetic correlations in large datasets of thyroid diseases, TH levels, and three common psychiatric disorders coinciding with thyroid dysfunction, that is, major depressive disorder (MDD), bipolar disorder (BIP), and ANX.

## Methods

### Data

This research was completed in accordance with the Declaration of Helsinki as revised in 2013. The U.K. biobank dataset was obtained through an application from Radboud University Medical Centre. This dataset is a large-scale resource for genetic, health, and lifestyle data.^22^ Subjects have been invited using patient registers. U.K. biobank is a sample of people of the United Kingdom. Individuals can voluntarily participate following invitation through patient's registration files at a general practitioner (almost everyone in United Kingdom is registered at a general practitioner [GP]).

Participants (*n* = 502,489, Supplementary Table S1) were recruited between March 2006 and October 2010 at baseline. There were 23 nonimaging and 4 imaging assessment sites for recruiting participants across the United Kingdom. Age of participants at baseline was in the range of 40–72 years (mean of 56.6 years and standard deviation of 8.1 years). A total of 409,529 subjects (81.5%) had “white British” ancestry based on a combination of self-declared ancestry and genomic principal components, with others declaring various ancestries spanning Asian or Asian British, Black or Black British, Chinese, mixed, and other groups.

U.K. biobank has institutional approval from the North West Multi-Centre Research Ethics Committee (MREC) as a Research Tissue Bank (RTB) approval. Researchers do not require separate ethical clearance and can operate under the RTB approval. The genome-wide summary statistics data were obtained from public repositories. These genome-wide summary data do not include individual or patient information and are generally released publicly without embargo following publication (Table 1).^19,20,23–26^

**Table 1.** Genome-Wide Association Study Summary Data Used in the Current Work

Phenotype	Class	Sample size
TSH normal range	Continuous	72,167
fT4 normal range	Continuous	72,167
TSH whole range	Continuous	119,715
Autoimmune thyroid disease	Categorical	755,406
Hypothyroidism^a^ (self-reported)	Categorical	361,141
Hyperthyroidism^a^ (self-reported)	Categorical	361,141
ANX	Categorical	83,566
BIP	Categorical	51,710
MDD	Categorical	480,359

^a^ Retrieved from https://github.com/Nealelab

ANX, anxiety disorder; BIP, bipolar disorder; fT4, free thyroxine; MDD, major depressive disorder; TSH, thyrotropin.

### Phenotypic correlation

We studied the co-occurrence of thyroid dysfunction and psychiatric disorders in 502,480 subjects of U.K. biobank. The clinical diagnosis of hyperthyroidism or hypothyroidism was recorded as *International Classification of Diseases* (ICD)-10 codes using inpatient medical records during a hospital stay (U.K. biobank data field 41202). More specifically, hypothyroidism was defined by either *Hypothyroidism, unspecified* (E039) or *autoimmune thyroiditis* (E063) codes. For hyperthyroidism, we considered *Thyrotoxicosis with diffuse goiter (*E050), toxic single thyroid nodule (E051), *Thyrotoxicosis with toxic multinodular goiter* (E052), and *Thyrotoxicosis, unspecified* (E059).

Subjects were excluded based on multiple ICD-10 codes associated with thyroid conditions (*n* = 2730 subjects), spanning malignant or benign neoplasm of thyroid gland (C73 and D34), carcinoma *in situ* of thyroid and other endocrine glands (D093), thyrotoxicosis from ectopic thyroid tissue (E053) factitial thyrotoxicosis (E054), other thyrotoxicosis (E058), acute, subacute, or drug-induced thyroiditis (E060, E061, and E064), chronic thyroiditis with transient thyrotoxicosis (E062) and other or unspecified chronic thyroiditis (E065, E069), congenital hypothyroidism with diffuse goiter (E030) and without goiter (E031), hypothyroidism due to medications and other exogenous substances (E032), postinfectious hypothyroidism (E033), acquired atrophy of thyroid (E034), myxedema coma (E035), other specified hypothyroidism (E038), and postprocedural hypothyroidism (E890). Subjects who reported using lithium (*n* = 503 subjects) or amiodarone (*n* = 456 subjects), which are known to affect thyroid function, were removed from the analysis. Subjects who underwent radiofrequency ablation of thyroid (*n* = 113) were also excluded from the analysis, as well as subjects who reported a history of both hypothyroidism and hyperthyroidism (*n* = 1178). In total, 4763 subjects were excluded from the analysis based on the above criteria, leaving 497,717 for further analysis.

For a broader definition of thyroid disease patients, and especially for including those patients who never received in-patient treatment and were diagnosed by general practitioners, we used self-reported diagnoses in U.K. biobank (data field 20002). We further used the self-reported medication field (data field 20003), which records those medications that individuals were actively taking at the time of verbal interview with U.K. biobank. For hypothyroidism, we considered medication codes for *levothyroxine sodium* (1141191044), *thyroxine sodium* (1140874852), *thyroxine product* (1140884516), and *sodium thyroxine* (1140910814). For hyperthyroidism, we considered *carbimazole* (1140874866), *propylthiouracil* (1140874776), and *propylthiouracil product* (1141157288).

In the current work, we considered three common psychiatric disorders, including MDD, BIP, and ANX. These phenotypes were extracted from various data fields and questionnaires similar to previous studies.^24–26^ For selecting MDD subjects, we combined data fields 2090 and 2100: *seen doctor (GP) or psychiatrist for nerves, anxiety, tension, or depression* and in-patient *ICD-10 codes (data field* 41202).

For ANX, we used data field 20544: *Mental health problems ever diagnosed by a professional, and* included codes for social anxiety or social phobia, any other phobia (e.g., disabling fear of heights or spiders), panic attacks, anxiety, nerves or generalized ANX, and agoraphobia. Using the same data field, subjects were removed from the anxiety group if they reported schizophrenia, mania, hypomania, bipolar or manic-depression, Asperger's or autism spectrum disorder, attention deficit or attention deficit and hyperactivity disorder and bulimia nervosa, psychological overeating or binge eating.

BIP subjects were selected using the same data field with codes referring to *mania, hypomania, bipolar or manic-depression*. Further details of these choices are available in previous works focusing on mood disorders in the U.K. biobank cohort.^27–29^

Due to the large sample size and strong statistical power, we decided to control for the confounding effects of age that may go beyond a linear trend.^30^ Therefore, the statistical association of thyroid disease with each of the three psychiatric disorders was assessed using logistic regression while controlling for the confounding effects of sex, age, age^2^, the interactions between sex × age and sex × age^2^, and smoking status.

### Global genetic correlation

The genetic correlations of TSH and free thyroxine (fT4) and thyroid diseases with ANX, MDD, and BIP were estimated using linkage disequilibrium (LD)-score regression (https://github.com/bulik/ldsc^31,32^) and the largest GWAS summary statistics available for each phenotype at the date of analysis. The GWASs of TSH and fT4 levels within the normal range were retrieved from the Thyroidomics meta-analysis.^19^ This meta-analysis included data from 22 independent cohorts, measuring TSH and fT4 using various assays in participants from the general population without known thyroid disease or treatment. The assays and reference ranges have been previously published as supplementary data.^19^

The GWAS summary statistics of TSH in the entire range (i.e., including healthy subjects and subjects affected by thyroid disease) was retrieved from a study by Zhou et al,^20^ as well as a recent GWAS of autoimmune thyroid disorders.^23^ We further considered recent GWASs of BIP,^25^ ANX,^24^ and MDD,^26^ which are available at the psychiatric genomics consortium portal (https://www.med.unc.edu/pgc^33^). GWAS data were filtered using minor allele frequency >0.01 and an imputation info score cutoff of 0.9. The GWASs of multiple phenotypes in U.K. biobank have been previously performed by Neale lab and generously released for third-party use. This dataset includes the GWASs of thousands of phenotypes related to lifestyle, general health, and medical history conditions (https://github.com/Nealelab/UK_Biobank_GWAS). We retrieved the SNP summary statistics of self-reported Hypothyroidism and Hyperthyroidism phenotypes from this repository (Table 1).

### Local genetic correlation

For those phenotype pairs demonstrating significant global genetic correlations, we subsequently tested their local genetic correlation using Local Analysis of Variant Annotation (LAVA^34^). This procedure divides the genome into 2495 local LD blocks and then estimates genetic correlations within each LD block. A local genetic correlation model could be fit to 275 LD blocks for the anxiety GWAS and 352 LD blocks for the major depression GWAS after overlapping each GWAS with the autoimmune thyroid disease GWAS. These multiple tests result in Bonferroni-corrected thresholds of 1.8 × 10^−4^ for ANX and 1.4 × 10^−4^ for MDD.

After identifying LD blocks of strong genetic correlation using LAVA, these “hotspots” were removed from the global genetic correlation analyses, to estimate the remaining genetic correlation excluding the local hotspots. This analysis was aimed at understanding whether the coheritability of thyroid disease and psychiatric disorders was driven by a small number of strong local genetic correlations or was rather distributed at many loci across the genome.

## Results

### Phenotypic correlations

**FIG. 1.**
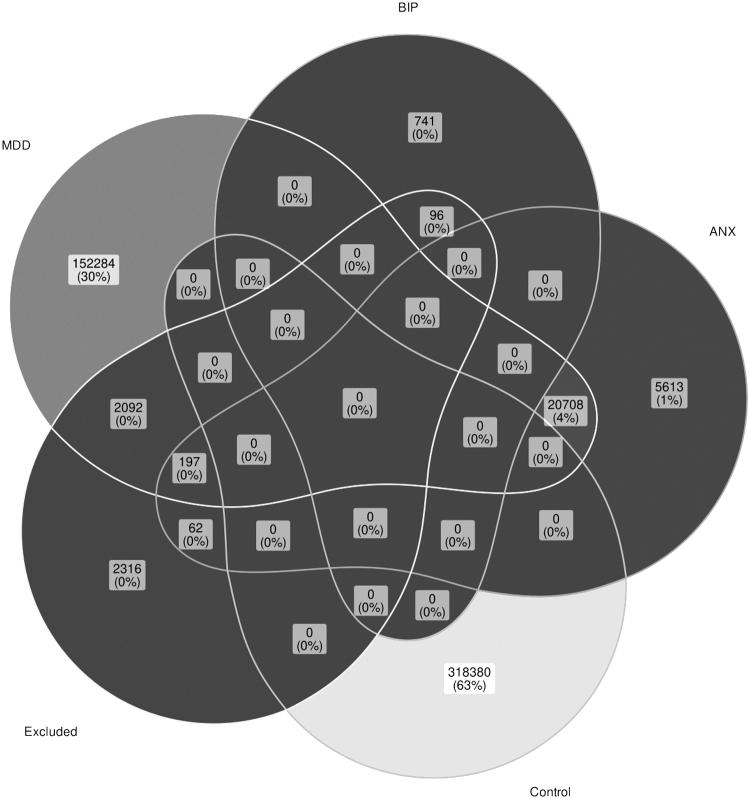
U.K. biobank subjects with various mood conditions. ANX, anxiety disorder; BIP, bipolar disorder; MDD, major depressive disorder.

**FIG. 2.**
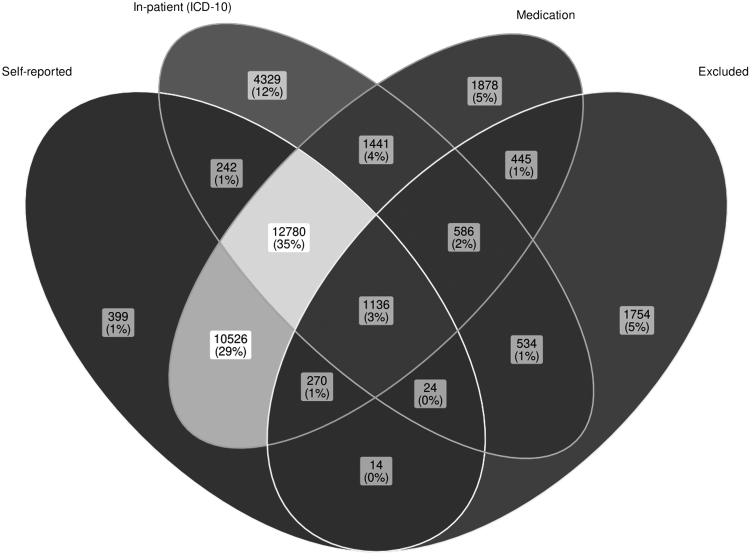
U.K. biobank hypothyroidism subjects based on self-report and in-patient ICD-10 data records and their medication status. *ICD, International Classification of Diseases.*

**FIG. 3.**
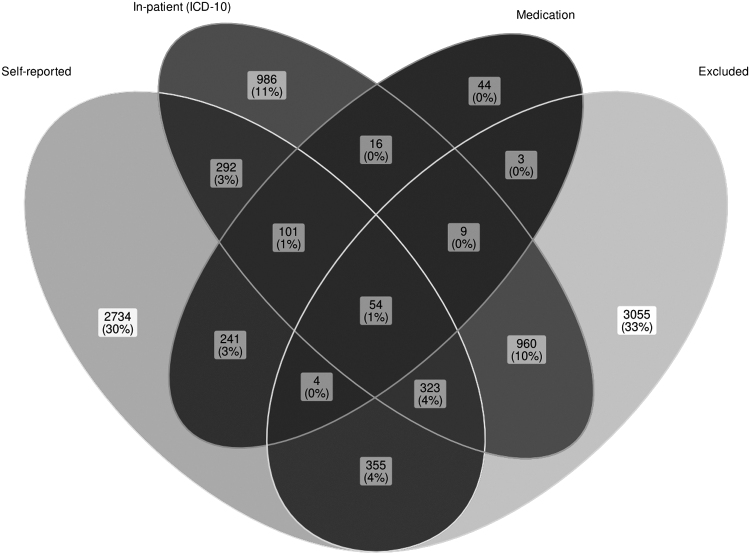
U.K. biobank hyperthyroidism subjects based on self-report and in-patient ICD-10 data records and their medication status.

First, we verified associations between thyroid dysfunction, mood, and ANXs in a large population-based cohort (U.K. Biobank), the results of which are shown in Table 2. The most common mood disorder was MDD (broad definition^26^) with a prevalence of 34.8% in the cohort, followed by ANX (5.3%) and BIP (0.15%, Fig. 1). The prevalence of hypothyroidism was 3.8% using in-patient ICD-10 records and 4.8% using self-reported diagnoses (Fig. 2). The prevalence of hyperthyroidism was 0.28% for in-patient ICD-10 records and 0.68% for self-reported diagnoses (Fig. 3). At the time of interview, ∼96% of self-reported hypothyroidism subjects indicated that they were actively receiving medications for hypothyroidism. In contrast, only ∼10% of self-reported hyperthyroidism subjects were actively taking medications for this condition. A total of 53,326 subjects (∼10.7%) reported a history of smoking. In the subsequent analyses, we corrected for smoking status in the regression models.

**Table 2.** Phenotypic Correlations Between Thyroid Dysfunction and Three Psychiatric Disorders

Phenotypes	Odds ratio	β	β SE	z-score	*p*
Hypothyroidism (in-patient ICD)/BIP	1.67	0.51	0.16	3.10	0.0019
Hypothyroidism (in-patient ICD)/MDD	1.48	0.39	0.02	25.64	5.00 × 10^−145^
Hypothyroidism (in-patient ICD)/ANX	1.05	0.05	0.03	1.56	0.12
Hypothyroidism (self-reported)/BIP	1.55	0.44	0.15	2.89	0.0038
Hypothyroidism (self-reported)/MDD	1.31	0.27	0.01	20.00	5.29 × 10^−89^
Hypothyroidism (self-reported)/ANX	1.16	0.15	0.03	5.41	6.22 × 10^−8^
Hyperthyroidism (in-patient ICD)/BIP	0.00	−12.04	173.51	−0.07	0.95
Hyperthyroidism (in-patient ICD)/MDD	1.17	0.16	0.06	2.89	0.0038
Hyperthyroidism (in-patient ICD)/ANX	1.17	0.16	0.11	1.46	0.15
Hyperthyroidism (self-reported)/BIP	0.84	−0.18	0.50	−0.35	0.72
Hyperthyroidism (self-reported)/MDD	1.11	0.10	0.04	2.93	0.0034
Hyperthyroidism (self-reported)/ANX	1.34	0.29	0.07	4.53	5.99 × 10^−6^

ICD, *International Classification of Diseases*; SE, standard error.

Considering in-patient ICD-10 records, hypothyroidism was positively associated with an increased risk of MDD and BIP. The strongest association was observed between hypothyroidism and BIP (OR = 1.67). Using the broader self-reported definition, hypothyroidism was associated with all three mood conditions at a significant level (Table 2).

When using in-patient ICD-10 recorded data, hyperthyroidism was associated with an increased risk of MDD. Using the broader self-reported hyperthyroidism designation slightly increased the strength of this association and further resulted in a significant association with ANX (*p* = 6 × 10^−6^, Table 2).

### Global genetic correlations

Next, we investigated global genetic correlations between thyroid traits and mood and ANXs, the results of which are shown in Table 3, along with the observed SNP-heritability estimates (h2-SNP). Among the traits, hyperthyroidism had very low SNP heritability (0.006) and was therefore not included in subsequent analyses.

**Table 3.** Global Genetic Correlation Between Thyroid Hormone Levels and Thyroid Diseases with Affective Disorders

	h2 SNP	ANX, r_g_ (*p*)	MDD, r_g_ (*p*)	BIP, r_g_ (*p*)
h2 SNP		0.08	0.07	0.35
Self-reported hypothyroidism (U.K. biobank)	0.05	0.17 (2.2 × 10^−^⁴)	0.17 (8.5 × 10^−^⁵)	0.02 (0.52)
TSH normal range	0.11	0.05 (0.43)	−0.05 (0.37)	−0.04 (0.42)
TSH entire range	0.10	0.02 (0.67)	−0.03 (0.55)	−0.06 (0.12)
fT4 normal range	0.15	−0.02 (0.71)	−0.04 (0.36)	−0.03 (0.55)
Hypo and hyperthyroid disease^a^	0.03	0.16 (6.7 × 10^−^⁶)	0.13 (2.7 × 10^−^⁴)	0.004 (0.89)

^a^ Retrieved from the summary statistics of an autoimmune thyroid disease GWAS by Saevarsdottir et al.^23^

GWAS, genome-wide association study.

We observed strong genetic correlations between hypo- and hyperthyroidism (using the summary statistics of an autoimmune thyroid disease GWAS^23^) and MDD (*r*_g_ = 0.13, *p* = 2.7 × 10^−^⁴) and ANX (*r*_g_ = 0.16, *p* = 6.7 × 10^−^⁶), but not with BIP. As (autoimmune) thyroid diseases include both autoimmune hypothyroidism, as well as Graves' disease, the results were validated using an independent GWAS on self-reported hypothyroidism in U.K. biobank (Table 3), concluding that the observed associations were predominantly driven by autoimmune hypothyroidism.

Next, to explore whether the observed genetic correlations with hypothyroidism are driven by TH levels (evaluated by a cross-section measurement) or autoimmunity (i.e., independent of TH levels), genetic correlations between TH levels, mood, and ANXs were tested. Despite higher SNP-heritability estimates for TSH and fT4, there were no significant genetic correlations between TSH or fT4, either in the normal or the entire (healthy and disease) range, and any of the mood and ANXs.

### Local genetic correlation

Finally, we carried out local genetic correlation analyses to refine the genomic regions underlying the observed global genetic correlations between hypothyroidism, mood, and ANXs. Strong local genetic correlation blocks were observed between autoimmune thyroid disease, ANX, and MDD at loci 6p22.1 and 6p22.2 (Tables 4 and 5), flanking the major histocompatibility complex (MHC) region. Another such “hotspot” of local genetic correlation was identified in chromosome 12, in a region containing multiple genes (RAP1B, NUP107, SLC35E3, MDM2, CPM, CPSF6, LYZ, YEATS4, FRS2, CCT2, LRRC10, BEST3; Tables 4 and 5).

**Table 4.** Top Local Genetic Correlations Between Autoimmune Thyroiditis and Anxiety Disorder

Locus	Chr	Start	Stop	SNP count	PCs	ρ	*p*
952	6	27261036	28666364	316	41	0.87	6.5 × 10^−^⁶
1804	12	68839662	70097805	459	135	1	0.00011
951	6	26396201	27261035	202	32	0.72	0.00060
267	2	59251997	60775066	626	178	−0.67	0.00071
932	6	6621938	7808323	519	179	−0.81	0.00077
2072	15	69089816	70767983	486	166	0.80	0.0015
2453	21	39252060	40480831	511	175	−0.69	0.0021
861	5	102903986	103788460	271	89	−0.77	0.0032
1559	10	79952997	81190573	529	172	−0.61	0.0035
1215	7	138755108	140217630	325	117	−0.75	0.0057
2485	22	41722788	42867927	277	59	0.84	0.0065
1391	9	15839404	16658377	413	141	−0.70	0.0097
1671	11	60515106	61717117	269	94	0.71	0.012
226	2	22429641	23538527	315	102	−0.86	0.012
203	2	1376546	1875060	186	72	−0.72	0.013
1576	10	98005995	99367800	466	125	−0.75	0.014
2022	14	95946793	97174314	539	165	−1	0.016
1059	6	131457165	132890694	404	123	0.61	0.016
35	1	36855140	38474036	514	137	0.73	0.018

**Table 5.** Top Local Genetic Correlations Between Autoimmune Thyroiditis and Major Depressive Disorder

Locus	Chr	Start	Stop	SNP count	PCs	ρ	*p*
951	6	26396201	27261035	294	42	0.77	2.7 × 10^−^⁶
952	6	27261036	28666364	591	60	0.77	2.8 × 10^−^⁵
961	6	31427210	32208901	221	61	0.51	0.00022
11	1	8580988	9475966	224	78	−1	0.00048
950	6	25684630	26396200	455	67	0.82	0.00089
960	6	31320269	31427209	19	15	0.55	0.0013
57	1	66778016	67761890	540	91	−1	0.0015
932	6	6621938	7808323	675	203	−0.82	0.0016
1301	8	64215359	66018204	531	108	0.70	0.0027
1804	12	68839662	70097805	581	152	0.92	0.0030
759	4	177129679	178314527	540	144	−0.74	0.0031
2336	19	29389091	30375792	416	115	−1	0.0035
332	2	141802665	142494980	378	88	1	0.0035
957	6	30715007	31106493	64	23	0.42	0.0038
966	6	32629240	32682213	2	2	1	0.0043
968	6	32897999	33194975	106	33	0.66	0.0054
478	3	65326752	66702084	514	170	−0.78	0.0071
1283	8	40974255	41725002	324	99	1	0.0079
2405	20	41351515	42185671	363	127	−0.92	0.0081

Contrary to our expectation, after excluding these “hotspots” of local genetic correlation, the calculated global genetic correlation remained high (ANX *p* = 4.2 × 10^−6^ and MDD *p* = 2.7 × 10^−4^), indicating that the observed genetic correlations are spread across the genome rather than localized to a few hotspots.

## Discussion

In the current study, we systematically analyzed the role of common genetic variants in the complex relationships between thyroid function, autoimmunity, and three common psychiatric disorders. We found strong genetic correlations between autoimmune hypothyroidism, MDD, and ANXs. Despite higher SNP-heritability estimates for thyroid function (TSH and fT4), there were no significant genetic correlations between TSH and fT4 and any of these psychiatric disorders. This may suggest that the genetically determined relationship between hypothyroidism and mood/ ANXs is not mediated through THs directly, but using pathways that underpin disease pathogenesis, such as (general) autoimmunity. This was further supported by our regional genetic correlation analyses, in which the top locus was the MHC region, which plays a central role in the pathogenesis of various autoimmune disorders, including autoimmune hypothyroidism.^35^

It has been known for long that inflammatory pathways play a central role in the pathogenesis of autoimmune hypothyroidism. However, in the last decade various studies have shown that inflammatory pathways also play a key role in the pathogenesis of many psychiatric disorders.^36–38^ Notably, studies on inflammatory biomarkers have been unable to detect markers that are specific for any particular psychiatric disorder, thereby suggesting genetic variation in autoimmunity to be a more general underlying phenomenon. Various biological treatment regimes, including pharmacotherapy and noninvasive brain stimulation with electroconvulsive therapy, indeed led to a reduction of pro-inflammatory markers, highlighting the role of inflammation in psychiatric vulnerability.^39,40^ Next to inflammatory pathways leading to both autoimmune hypothyroidism and increased psychiatric vulnerability, coinciding autoimmune disorders might also for other reasons than shared inflammatory pathways be responsible for the observed relationships between thyroid and psychiatric disorders.

Indeed, it has been shown that autoimmune hypothyroidism coincides with several other autoimmune conditions, such as rheumatoid arthritis, celiac disease, type 1 diabetes, premature ovarian failure, and adrenal insufficiency.^41^ These comorbidities have also been associated with various psychiatric diseases, including MDD and ANXs. Therefore, future studies in well-phenotyped cohorts should assess which part of the observed associations between hypothyroidism and psychiatric disorders is actually driven by these other autoimmune comorbidities. Furthermore, one of the several mechanistic hypotheses underlying the coincidence of multiple autoimmune conditions is that various tissues share the same epitopes, to which autoantibodies can cross-react.^42^ In this context, Benvenga et al^43^ recently showed an interesting amino acid sequence homology between thyroid autoantigens and central nervous system proteins, including TPO, autoantibodies to which are a characteristic biomarker for Hashimoto's hypothyroidism.

One way to investigate the mechanistic relationship between hypothyroidism and mood disorders is to perform genetic correlation analyses on TPO antibodies and mood disorders. To date, the GWAS of TPO antibodies has not shown a strong heritable signal (h2_SNP_ <0.002), probably due to a combination of limited statistical power and the fact that it was performed in population-based cohorts after excluding individuals on thyroid medication, effectively excluding most of the subjects with diagnosed Hashimoto's hypothyroidism.^44^ Therefore, future larger GWASs of (thyroid) autoimmune biomarkers and conditions will help elucidating whether the thyroid-mood axis is predominantly mediated through TH levels or pathways involved in (auto)immunity.

Irrespective of the exact underlying mechanisms, our findings also have clinical relevance. Based on the well-known clinical correlations, thyroid dysfunction is among the first somatic disorders to be ruled out in a patient with a newly diagnosed psychiatric disorder. Various studies have investigated whether correction of milder forms of thyroid dysfunction (including subclinical hypothyroidism) improves depressive and anxiety-related symptoms, with disappointing results.^45,46^ Our finding that the genetically determined relationship between hypothyroidism and mood/ANXs is not mediated through THs, but more likely by (general) autoimmunity, is a plausible explanation for this lack of effects and supports a restrictive attitude toward treating mild thyroid dysfunction in psychiatric patients.

Besides psychiatric disorders, milder affective and anxiety related complaints are frequently reported in hypothyroid patients. Unfortunately, these complaints persist in 10–15% of levothyroxine treated patients despite normalization of their TH levels, which is among the largest knowledge gaps in endocrinology.^47^ Our findings may not suggest direct psychiatric vulnerability of Hashimoto's hypothyroidism patients, and may highlight that this comorbidity is partly mediated through autoimmunity and not thyroid function, which would be a plausible explanation as to why these patients have persisting complaints despite the normalization of their TH levels after initiating thyroid medication.

Our combined phenotypic and genetic analyses across multiple psychiatric and thyroid traits allowed us to better understand their shared biological underpinnings, more so than we would have in a purely phenotypic analysis. While our evidence for genetic overlap strongly encourages further research into shared molecular mechanisms, the approaches we used remain observational and do not directly exclude alternative causal explanations. For example, heritable lifestyle factors (such as diet and physical activity) are shared predisposing factors to both psychiatric disorders and thyroid diseases, so these could be contributing mediating factors in addition to any directly mediating, purely molecular inflammation pathways.^48–52^ Future research using Mendelian randomization^53^ and longitudinal designs is warranted to better inform on the different possible causal mechanisms.

Our local genetic correlation analysis identified two loci as potential mediators of the genetic overlap between thyroid and mood/ANXs. Notably, one of these two regions maps to 6p22.1. Common variants in this region have been previously implicated in schizophrenia.^54,55^ This locus flanks the MHC genes and GABBR1, the latter coding for a GABAergic synaptic receptor. In addition, this locus is a genetic correlation “hub” across multiple, mostly (auto)immune-related clinical conditions.^34^

Our genetic analysis was made possible by the recent surge in well powered GWAS data from very large sample sizes and consortia, both in psychiatry and endocrinology. The resultant power and the fact that our genetic correlations are based on independent samples strengthen the present study. However, a limitation of using existing GWAS statistics is that they differ in statistical power, and consequently, the genetic correlations are not directly comparable across traits. Despite being based on larger sample sizes than the autoimmune hypothyroidism analyses, no genetic correlation was found between TSH/fT4 and psychiatric disorders. This strengthens the finding that the observed association between autoimmune hypothyroidism and psychiatric disorders is for an important part autoimmune related.

It is difficult to know whether the strong phenotypic correlation we found between BIP and hypothyroidism is not driven by genetic variation at all or whether it is due to lower statistical power in the BIP GWAS data we used. Nevertheless, GWAS data for MDD and anxiety were well powered and provided robust results that were consistent across two independent GWASs of thyroid disease. Similarly, differences in prevalence across different diseases is a limitation of the phenotypic correlation analysis, especially in a population-based sample like U.K. Biobank. The higher prevalence for hypothyroidism and major depression led to well powered phenotypic correlation analyses and conclusive results.

The phenotypic correlations we observed between hypo- and hyperthyroidism with mood disorders did not replicate the exact genotypic correlation patterns. A potential explanation for this contrast may be that, phenotypically, environmental exposures and their correlation with genetic factors “dilute” the genetic overlap. Similar to many polygenic traits, the SNP heritability of various mood disorders (h2_SNP_ = 0.07–0.35) explain a fraction of total phenotypic variability. Further research, especially using longitudinal studies on environmental factors and trait-modifying variables and their association with genotypes, is needed to elucidate such divergence in genotype and phenotype correlations.

We cannot exclude a multitude of factors that confound our (and almost every) genetic correlation analyses, such as environmental factors, nonrandom (assortative) mating, gene-by-environment correlations (e.g., diet), and sampling bias. It is noteworthy that individuals with mood disorders are more likely to be investigated for thyroid dysfunction than the general population. Some of such individuals are ultimately diagnosed with mild or subclinical thyroid dysfunction without evidence of intrinsic thyroid disease, and this can be a potential source of (sampling) bias in our work. However, when, for example, focusing on hypothyroidism, 5% of patients with depression in U.K. Biobank were found to have hypothyroidism. By definition, ∼2.5% of the general population have an increased TSH level. So even if all depressed patients with an increased TSH would have been treated (for supposed hypothyroidism), this does not explain the 5%.

Furthermore, we used biobank-derived data for parts of our phenotypic and genotypic correlation analyses. Although sample sizes of large datasets such as U.K. biobank provide strong statistical power, due to costs and practicalities, these data are inherently less detailed with regard to clinical characteristics and comprehensive usage of standard diagnostic criteria. In this regard, our study is prone to the noise of broadly defined mood disorder phenotypes and (sub)clinical conditions that may not fulfill standard diagnostic criteria. However, previous works have shown that clinical phenotypes are strongly genetically correlated with self-declared and broadly defined “proxy” phenotypes, for example, in GWAS applications, even when recorded in subjects' parents and in depression phenotypes in U.K. biobank.^23,27,56^ Taken together, we believe that the benefits of performing preliminary analyses of such broadly defined phenotypes in large biobank-based samples outweigh such limitations.

As a sample of people of the United Kingdom, both U.K. biobank and genome-wide data used in this report are mostly representative of the European ancestry, specifically those self-reported and genetically clustered as “white British” populations. Due to this, caution must be taken with regards to the generalizability of our results to other ancestries, and further diversification of the future works is warranted. For the genetic correlation analyses, genome-wide summary statistics was used, so we don't have access to individual level data. Lack of access to in-depth individual-level GWAS data is a limitation of our work.

In conclusion, our results indicate that, rather than TH itself, other explanations (such as autoimmunity) play a key role in the genetically determined relationships between hypothyroidism and mood/ANXs. Next to providing valuable clinical insights into the potential benefits of treating milder forms of thyroid dysfunction, these findings warrant further research into the autoimmune mechanisms underpinning these common coexisting diseases that may lead to a shift in personalizing treatment in the future.

## Supplementary Material

Supplemental data
