# Supplementary material for: Exploring the Genetic Link Between Thyroid Dysfunction and Common Psychiatric Disorders: A Specific Hormonal or a General Autoimmune Comorbidity
Source: Thyroid. 2023 Feb 14;33(2):159–68. doi: 10.1089/thy.2022.0304 (PMC10133968; doi:10.1089/thy.2022.0304)
Supplement: Supplemental data [file Supp_TableS1.docx]

Supplementary Table S1. UK biobank subject demographics and excluded individuals.

| Phenotype | #Subjects |
| --- | --- |
| Age | 56.6 ± 8.1y (mean ± standard deviation) |
| Sex | 269,940 female |
| Caucasian ancestry | 409,529 |
| Hypothyroidism (ICD-10) | 18,792 |
| Hyperthyroidism (ICD-10) | 1,395 |
| Hypothyroidism (self-reported) | 23,947 |
| Hyperthyroidism (self-reported) | 3,368 |
| Medication for hypothyroidism | 26,625 |
| Medication for hyperthyroidism | 402 |
| Anxiety | 26,321 |
| Major depression | 172,992 |
| Bipolar disorder | 741 |
| Smoking | 53,326 |
| Thyroid-related comorbidities (excluded) | 2,730 |
| Radiofrequency ablation of thyroid (excluded) | 113 |
| Positive history of both hypo- and hyper-thyroidism (excluded) | 1,178 |
| Amiodarone medication (excluded) | 456 |
| Lithium medication (excluded) | 503 |
| Total subjects | 502,489 |
| Total subjects included in the analysis | 497,726 |
